# Feasibility and Safety of Same-Day Discharge Following Elective Mitral Transcatheter Edge-to-Edge Repair

**DOI:** 10.1016/j.jacadv.2026.102656

**Published:** 2026-04-25

**Authors:** Mahmoud Ismayl, Andrew M. Goldsweig, Mohamad Alkhouli, Mackram F. Eleid, Charanjit S. Rihal, Mayra Guerrero

**Affiliations:** a Department of Cardiovascular Medicine, Mayo Clinic, Rochester, Minnesota, USA; b Department of Cardiovascular Medicine, Baystate Medical Center, Springfield, Massachusetts, USA; c Department of Cardiovascular Medicine, University of Massachusetts-Baystate, Springfield, Massachusetts, USA

**Keywords:** mitral transcatheter edge-to-edge repair, M-TEER, outcomes, readmissions, same-day discharge

## Abstract

**Background:**

Data on the feasibility and safety of same-day discharge (SDD) following mitral transcatheter edge-to-edge repair (M-TEER) remain limited.

**Objectives:**

The objective of the study was to evaluate the contemporary trends and outcomes of SDD following M-TEER.

**Methods:**

We reviewed the Nationwide Readmissions Database (2016-2022) to compare SDD following elective M-TEER with next-day discharge (NDD). The primary outcome was unplanned all-cause readmissions within 90 days. Secondary outcomes included 90-day heart failure (HF) readmissions and index hospitalization costs. Readmissions were compared using a Cox proportional hazards regression model.

**Results:**

A total of 39,804 weighted hospitalizations for M-TEER were included, of which 1.2% involved SDD, 69.0% NDD, and 29.8% second- or third-day discharge (ScD/TDD). From 2016 to 2022, the proportion of SDD and NDD increased from 0.4% to 2.4% and 47.9% to 76.7%, respectively, whereas ScD/TDD decreased from 51.7% to 20.9% (all p_trend_<0.001). SDD was associated with a similar risk of 90-day all-cause readmission (14.8% vs 12.5%; adjusted HR: 1.25; 95% CI: 0.90-1.74) and HF readmission (4.4% vs 3.7%; adjusted HR: 1.22; 95% CI: 0.67-2.22) and significantly lower index hospitalization costs ($38,029 vs $43,319; *P* = 0.03) compared to NDD. ScD/TDD was associated with a higher risk of 90-day HF readmission—but not all-cause readmission—and higher index hospitalization costs ($49,648 vs $43,319; *P* < 0.001) compared to NDD.

**Conclusions:**

SDD following M-TEER is uncommon but associated with a similar risk of 90-day readmission and lower index hospitalization costs compared to NDD. Further research is warranted to identify optimal patient selection criteria for SDD after M-TEER.

Mitral transcatheter edge-to-edge repair (M-TEER) is an established, minimally invasive treatment for patients with severe symptomatic primary (degenerative) mitral regurgitation (MR) who are at high or prohibitive surgical risk and in selected patients with secondary (functional) MR who remain symptomatic despite guideline-directed medical therapy.^1^^,^^2^ As the adoption of M-TEER increases, optimizing postprocedural care pathways has become a focus for improving health care efficiency, reducing costs, and enhancing patient satisfaction.

Same-day discharge (SDD) following structural heart interventions has garnered increasing attention in recent years.^3^ Several studies have shown that SDD is feasible and safe in selected patients undergoing transcatheter aortic valve replacement (TAVR)^4^, ^5^, ^6^ or left atrial appendage occlusion (LAAO),^7^^,^^8^ with short-term outcomes comparable to those of overnight or longer admissions. Although there is robust evidence supporting SDD following TAVR and LAAO, data on SDD after M-TEER remain limited. With ongoing advancements in device technology and rising procedural experience, the safety profile of M-TEER has significantly improved in contemporary practice.^9^ According to the most recent data from the Society of Thoracic Surgeons/American College of Cardiology Transcatheter Valve Therapy (STS/ACC TVT) Registry, the median length of stay (LOS) following M-TEER in 2019 was 1 day (IQR: 1-4 days), indicating that over half of patients were discharged on the next day or possibly sooner.^9^ These findings suggest that SDD may be a feasible strategy in appropriately selected patients following M-TEER.

To date, the literature on SDD following M-TEER is limited to a single case series (n = 6)^10^ and one single-arm retrospective cohort study (n = 82).^11^ The latter, conducted at the University of Texas MD Anderson Cancer Center, demonstrated the feasibility of SDD in 82 carefully selected patients who underwent successful MitraClip (Abbott) implantation under moderate sedation between February 2019 and April 2020. These preliminary findings suggest that SDD may be a safe and feasible strategy in selected M-TEER patients and underscore the need for larger, multicenter studies to validate its broader applicability. Our study is the first to evaluate comprehensively the feasibility and safety of SDD following M-TEER using a nationally representative dataset, comparing outcomes with next-day and later discharge strategies.

## Methods

### Data source and ethics statement

Hospitalization data were abstracted from the Nationwide Readmissions Database (NRD), which is part of the Healthcare Cost and Utilization Project (HCUP) family of databases sponsored by the Agency for Healthcare Research and Quality.^12^ The NRD is the largest publicly available, fully deidentified, all-payer inpatient health care readmission database in the United States. The NRD is compiled from billing data submitted by hospitals to statewide organizations across the United States and has reliable and verified patient linkage numbers that can be used to track patients across hospitals within each state and calendar year while adhering to strict privacy guidelines. The NRD includes approximately 18 million unweighted hospitalizations each year with a diverse patient population, growing from 27 states in 2016 to 30 states in 2022 (Supplemental Table 1).^12^ When weighted, the NRD extrapolates to the national level, representing approximately 35 million hospitalizations each year. The unweighted sample represents approximately 50% of all U.S. hospitalizations. The NRD contains both patient- and hospital-level information. Up to 40 discharge diagnoses and 25 procedure codes are collected for each patient using International Classification of Diseases-10th Revision (ICD-10) codes.^13^ The NRD captures all admissions and readmissions with nationally representative weighting, allowing the analysis of readmission rates. Each patient is assigned a unique identifier code using the variable “NRD_VistLink” to track patients within a calendar year. The NRD days-to-event variable (“NRD_DaysToEvent”) is used to capture readmissions within a calendar year but not across different years.^12^ The design and methodology of the NRD have been described previously.^14^, ^15^, ^16^, ^17^, ^18^ This study followed the STROBE (Strengthening the Reporting of Observational Studies in Epidemiology) reporting guideline (Supplemental Table 2)^19^ and was exempt from the requirements of the Mayo Clinic Institutional Review Board because the NRD is a fully deidentified, HIPAA-compliant database that is publicly available from the HCUP website (www.hcup-us.ahrq.gov).

### Study population and patient selection

**Figure 1:**
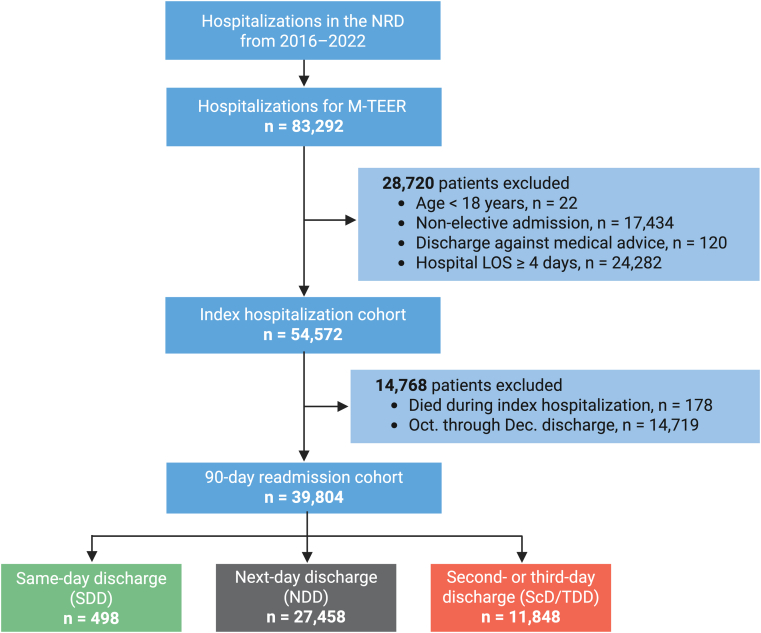
**Study Flow Diagram Showing Inclusion and Exclusion Criteria** Hospitalization counts represent national-level estimates. LOS = length of stay; M-TEER = mitral transcatheter edge-to-edge repair; NRD = Nationwide Readmissions Database.

The NRD was reviewed from January 2016 through December 2022 to identify hospitalizations in which adult patients (age ≥18 years) underwent M-TEER (*ICD-10*, Procedure Coding System 02UG3JH, 02QG3ZE, 02QG3ZZ, 02UG3JZ, 02UG37E, 02UG37Z, 02UG38E, 02UG38Z, 02UG3JE, 02UG3KE, 02UG3KZ, 02UG47E, 02UG47Z, 02UG48E, 02UG48Z, 02UG4JE, 02UG4JZ, 02UG4KE, and 02UG4KZ in any procedural field). Exclusions included hospitalizations involving patients aged <18 years, those discharged against medical advice, nonelective admissions, and those with a hospital LOS of ≥4 days. For hospitalizations that met the inclusion criteria, patients were stratified into 3 cohorts based on timing of hospital discharge: SDD, next-day discharge (NDD), and second- or third-day discharge (ScD/TDD) (Figure 1). SDD was defined as a LOS of 0 days in the NRD, corresponding to discharge on the same calendar day of admission. A complete list of *ICD-10* diagnosis and procedure codes used in this study is presented in Supplemental Table 3.

Per HCUP-NRD recommendations, when evaluating 90-day readmissions, we excluded hospitalizations in which the patient died during the index hospitalization as such patients have no risk of readmission, as well as any discharge occurring after September 30 of each calendar year because the NRD only tracks readmissions within a single calendar year (Figure 1).^12^ Therefore, to enable a full 90-day follow-up for all discharges, we only used data from January 1st to September 30th of each year for the analysis on 90-day readmissions. For patients who had multiple 90-day readmissions, only the first readmission was included in the analysis. The NRD does not provide data on out-of-hospital deaths, and therefore, patients who died at home within 90 days would be counted as patients without a readmission within 90 days.

### Study outcomes

Temporal trends in SDD, NDD, and ScD/TDD following M-TEER were reported. The primary outcome was unplanned all-cause readmissions within 30 and 90 days of discharge. Secondary outcomes included unplanned heart failure (HF) readmissions within 30 and 90 days and total costs of the index hospitalization and readmissions (inflation adjusted to 2022 U.S. dollars).^20^ Charge-to-cost ratio files were used to convert charges to costs at the individual hospital level. Independent predictors of SDD and 90-day all-cause readmission were also evaluated.

### Statistical analysis

Descriptive statistics were presented as counts and percentages for categorical variables and as medians with IQRs for continuous variables. Categorical variables were compared using the Pearson chi-square test or Fisher exact test as appropriate. Continuous variables were compared using the Mann-Whitney *U* test for 2-group comparisons and the Kruskal-Wallis test for comparisons involving 3 groups.

Temporal trends in SDD, NDD, and ScD/TDD were analyzed using linear regression. The probability of 30- and 90-day readmission, stratified by timing of hospital discharge, was graphically displayed using the Kaplan-Meier method and was compared using the log-rank test. To estimate the adjusted HR for 30- and 90-day readmissions for SDD and ScD/TDD vs NDD, a multivariable Cox proportional hazards regression model was generated using the following variables: age, sex, primary payer, median income quartile by zip code, hospital location (urban/rural) and teaching status, number of hospital beds, Elixhauser and Charlson Comorbidity Index scores, relevant comorbidities, procedural complications, and discharge disposition (Supplemental Table 4). Adjustment variables were selected a priori based on their clinical significance and their likely influence on readmissions. Estimated adjusted HRs were reported with corresponding 95% CIs. To further minimize the risk of potential confounding and selection bias, 2 sensitivity analyses were conducted: 1 excluding hospitalizations involving any procedural complication, and the other excluding hospitalizations in which patients were discharged to a short-term hospital or a skilled nursing/intermediate care facility. Propensity score matching methodology was also performed to match SDD and NDD patients in a 1:1 ratio. Propensity scores were derived from multivariable logistic regression predicting SDD using potential confounders (Supplemental Table 5). Nearest-neighbor matching with a caliper of 0.2 SDs of the logit of the propensity score was applied. Covariate balance before and after propensity score matching was assessed using standardized mean differences. Overlap and distribution of propensity scores between SDD and NDD groups were evaluated using kernel density plots of the propensity score before and after matching.

To account for the possibility of unobserved out-of-hospital deaths, we performed a sensitivity analysis assuming a 1.2% 30-day out-of-hospital mortality rate based on the STS/ACC TVT Registry.^21^ Hypothetical deaths were randomly assigned among patients without readmission, and both unadjusted and adjusted Cox regression models were re-estimated to evaluate robustness of the findings.

The multivariable Cox regression model was used to determine independent predictors of 90-day all-cause readmission using the same variables listed previously (Supplemental Table 4). The assumptions of Cox proportional hazards regression were assessed both graphically and formally. Graphical assessment was performed using log-log survival plots, which showed approximately parallel curves for all covariates, suggesting that the proportional hazards assumption was satisfied. Formal testing was conducted using Schoenfeld residuals, both globally and for individual covariates, with no significant correlation observed between residuals and time (all *P* > 0.05), confirming that the proportional hazards assumption was appropriate. A multivariable logistic regression model was used to determine independent predictors of SDD using variables listed in Supplemental Table 4.

Complete data were available for all variables except for primary payer (missing 0.1%) and median household income quartile by zip code (missing 0.8%). Patterns of missingness were sparse and not clustered in specific hospitalizations. Little’s test for Missing Completely at Random indicated no evidence of systematic missingness (*P* = 0.45). Given the minimal proportion of missing values and their distribution, missing data were handled with complete case analysis.

For all statistical analyses, a 2-tailed *P* < 0.05 was considered statistically significant. Given the large sample size, not all statistically significant *P* values represent clinically significant differences and therefore require careful interpretation. All statistical analyses were performed using Stata (version 17; StataCorp) software and R software for Statistical Computing (version 4.3; R Foundation for Statistical Computing), accounting for the NRD sampling design, and were weighted using sampling weights provided with the NRD to estimate national-level effects per HCUP-NRD recommendations similar to prior studies.^12^^,^^22^, ^23^, ^24^, ^25^, ^26^, ^27^

## Results

### Patient and hospital characteristics

From January 2016 through December 2022, a total of 39,804 weighted hospitalizations in the NRD met the inclusion criteria, of which 498 (1.2%) involved SDD, 27,458 (69.0%) NDD, and 11,848 (29.8%) ScD/TDD (Figure 1).

Patients discharged on the same day (SDD) were more frequently hospitalized in urban teaching hospitals with greater bed capacity. Compared to patients with NDD or ScD/TDD, those in the SDD group were more likely to have peripheral artery disease and a history of stroke or transient ischemic attack, coronary artery bypass grafting, or pre-existing implantable cardioverter-defibrillator or permanent pacemaker. In contrast, SDD patients were less likely to have renal failure, hemodialysis dependence, or chronic pulmonary disease. Patients in the ScD/TDD group had higher rates of coagulopathy compared to the NDD and SDD groups. Socioeconomic differences were also observed: a greater proportion of SDD patients resided in neighborhoods within the lowest quartile of median household income compared to NDD and ScD/TDD patients.

Procedural complications varied by discharge timing. Compared to NDD patients, those in the SDD group had lower rates of in-hospital complications including stroke, major bleeding, need for blood transfusion, vascular complications, and acute kidney injury. In contrast, ScD/TDD patients experienced higher rates of these complications.

Discharge destinations also differed by discharge timing. Compared to NDD patients, SDD patients were more often discharged home without services (480/498 [96.4%] vs 25,344/27,458 [92.3%]), whereas ScD/TDD patients were more frequently discharged to home health care (2,583/11,848 [21.8%] vs 1,894/27,458 [6.9%]) or skilled nursing/intermediate care facilities (367/11,848 [3.1%] vs 220/27,458 [0.8%]) (all *P* < 0.001). Baseline characteristics, procedural complications, and discharge dispositions stratified by timing of hospital discharge are shown in Table 1.

**Table 1:** Baseline Characteristics Stratified by Timing of Discharge

	SDD (n = 498)	NDD (n = 27,458)	ScD/TDD (n = 11,848)	*P* Value
Demographic characteristics				
Age, y	78 (71-84)	79 (73-85)	78 (69-84)	<0.001
18-64	54 (10.8)	2,773 (10.1)	2,074 (17.5)	<0.001
65-74	126 (25.3)	5,657 (20.6)	2,393 (20.2)	
75-84	212 (42.6)	12,081 (44.0)	4,431 (37.4)	
85+	106 (21.3)	6,947 (25.3)	2,950 (24.9)	
Biological sex				
Male	302 (60.6)	15,515 (56.5)	6,576 (55.5)	0.18
Female	196 (39.4)	11,943 (43.5)	5,272 (44.5)	
Primary payer				
Medicare	420 (84.3)	23,882 (87.0)	9,595 (81.0)	<0.001
Medicaid	16 (3.2)	681 (2.5)	247 (2.1)	
Private insurance	52 (10.4)	2,328 (8.5)	1,740 (14.7)	
Self-pay	0 (0)	131 (0.5)	116 (1.0)	
Other	<11 (<2.2)^a^	406 (1.5)	140 (1.2)	
Income quartile^b^				
I	121 (24.5)	5,716 (21.0)	2,238 (19.1)	0.01
II	113 (22.9)	6,650 (24.4)	2,795 (23.8)	
III	128 (25.9)	7,419 (27.2)	3,079 (26.2)	
IV	132 (26.7)	7,473 (27.4)	3,636 (30.9)	
Hospital characteristics				
Location/teaching status				
Urban nonteaching	<11 (<2.2)^a^	2,334 (8.5)	936 (7.9)	<0.001
Urban teaching	488 (98.0)	24,904 (90.7)	10,841 (91.5)	
Nonurban hospital	<11 (<2.2)^a^	220 (0.8)	71 (0.6)	
Bed size^c^				
Small	14 (2.8)	1,510 (5.5)	391 (3.3)	<0.001
Medium	74 (14.9)	5,162 (18.8)	2,903 (24.5)	
Large	410 (82.3)	20,786 (75.7)	8,554 (72.2)	
Clinical characteristics				
Elixhauser Comorbidity Index	5 (4-6)	5 (4-6)	5 (4-7)	<0.001
Charlson Comorbidity Index	2 (1-4)	2 (1-4)	2 (1-4)	0.55
0	36 (7.2)	1,730 (6.3)	1,303 (11.0)	<0.001
1	112 (22.5)	6,727 (24.5)	2,618 (22.1)	
2	108 (21.7)	5,711 (20.8)	2,145 (18.1)	
≥3	242 (48.6)	13,290 (48.4)	5,782 (48.8)	
Diabetes mellitus	134 (26.9)	6,672 (24.3)	2,832 (23.9)	0.51
Hypertension	410 (82.4)	22,900 (83.4)	9,715 (82.0)	0.20
Dyslipidemia	322 (64.7)	17,765 (64.7)	7,476 (63.1)	0.10
Nicotine/tobacco use	208 (41.8)	10,599 (38.6)	4,407 (37.2)	0.09
Alcohol abuse	<11 (<2.2)^a^	302 (1.1)	154 (1.3)	0.42
Drug abuse	<11 (<2.2)^a^	165 (0.6)	83 (0.7)	0.36
Obesity	70 (14.1)	3,213 (11.7)	1,505 (12.7)	0.23
Coronary artery disease	290 (58.2)	16,694 (60.8)	6,907 (58.3)	0.11
Peripheral artery disease	142 (28.5)	6,700 (24.4)	2,725 (23.0)	0.03
Atrial fibrillation/flutter	282 (56.6)	15,898 (57.9)	6,860 (57.9)	0.92
Congestive heart failure	424 (85.1)	23,312 (84.9)	9,490 (80.1)	<0.001
Renal failure	156 (31.3)	9,089 (33.1)	4,194 (35.4)	0.006
Dialysis dependent	<11 (<2.2)^a^	686 (2.5)	379 (3.2)	0.01
Liver disease	<11 (<2.2)^a^	604 (2.2)	237 (2.0)	0.72
Chronic pulmonary disease	90 (18.1)	6,260 (22.8)	2,903 (24.5)	0.004
Obstructive sleep apnea	76 (15.3)	3,762 (13.7)	1,552 (13.1)	0.41
Coagulopathy	22 (4.4)	1,236 (4.5)	1,208 (10.2)	<0.001
Cancer	18 (3.6)	796 (2.9)	332 (2.8)	0.74
Malnutrition	<11 (<2.2)^a^	110 (0.4)	107 (0.9)	<0.001
Dementia	<11 (<2.2)^a^	522 (1.9)	296 (2.5)	0.02
Depression	40 (8.0)	1,812 (6.6)	877 (7.4)	0.15
Previous history				
Myocardial infarction	82 (16.5)	4,311 (15.7)	1,742 (14.7)	0.18
Stroke/TIA	66 (13.3)	3,158 (11.5)	1,185 (10.0)	0.004
Cardiac arrest	<11 (<2.2)^a^	275 (1.0)	142 (1.2)	0.46
PCI	84 (16.9)	5,382 (19.6)	2,251 (19.0)	0.39
CABG	124 (24.9)	5,354 (19.5)	1,931 (16.3)	<0.001
ICD	84 (16.9)	3,624 (13.2)	1,291 (10.9)	<0.001
PPM	62 (12.5)	3,103 (11.3)	1,173 (9.9)	0.02
Procedural complications				
Stroke	0 (0)	27 (0.1)	95 (0.8)	<0.001
Major bleeding	0 (0)	55 (0.2)	107 (0.9)	<0.001
Need for blood transfusion	0 (0)	192 (0.7)	486 (4.1)	<0.001
Vascular complications	0 (0)	275 (1.0)	284 (2.4)	<0.001
Acute kidney injury	<11 (<2.2)^a^	467 (1.7)	746 (6.3)	<0.001
Discharge disposition				
Routine	480 (96.4)	25,344 (92.3)	8,886 (75.0)	<0.001
Transfer to short-term hospital	0 (0)	0 (0)	12 (0.1)	
Transfer to SNF or ICF	<11 (<2.2)^a^	220 (0.8)	367 (3.1)	
Home health care	16 (3.2)	1,894 (6.9)	2,583 (21.8)	

Values are median (IQR) or n (%). Two authors (M.I. and A.M.G.) independently verified the International Classification of Diseases-10th Revision (ICD-10) codes that corresponded to each comorbidity (Supplemental Table 3), and any disagreements in inclusion or exclusion of *ICD-10* codes were discussed with a third author (M.G).

CABG = coronary artery bypass grafting; ICD = implantable cardioverter-defibrillator; ICF = intermediate care facility; NDD = next-day discharge; PCI = percutaneous coronary intervention; PPM = permanent pacemaker; ScD/TDD = second- or third-day discharge; SDD; same-day discharge; SNF = skilled nursing facility; TIA = transient ischemic attack.

a Cell counts <11 are not reportable per HCUP guidelines.

b Estimated median household incomes are zip code–specific, updated annually, and classified into 4 quartiles indicating the poorest to wealthiest populations.

c Bed-size categories are based on inpatient beds and are specific to the hospital’s location and teaching status. A more detailed explanation of all the variables in the NRD, including the specific dollar amounts in each category of median household income and the number of hospital beds in each category, is available online (https://hcup-us.ahrq.gov/db/nation/nrd/nrddde.jsp).

### Trend analysis

**Figure 2:**
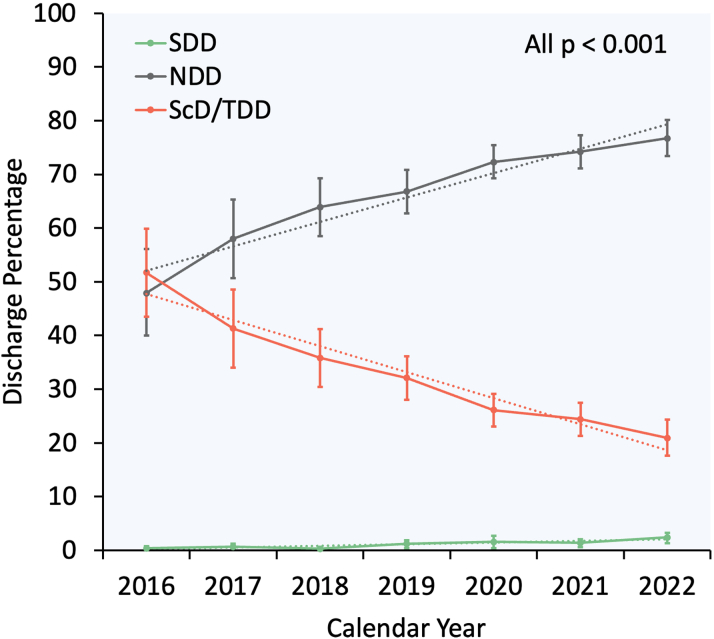
**Year-Over-Year Trend in SDD, NDD, and ScD/TDD Following M-TEER in the United States From 2016 to 2022** Error bars represent 95% CIs. Dotted lines represent linear trends. NDD = next-day discharge; ScD/TDD = second- or third-day discharge; SDD = same-day discharge; other abbreviation as in Figure 1.

From 2016 to 2022, the proportion of SDD and NDD increased from 0.4% to 2.4% and 47.9% to 76.7%, respectively, whereas ScD/TDD decreased from 51.7% to 20.9% (all p_trend_<0.001) (Central Illustration). Annual trends for SDD, NDD, and ScD/TDD following M-TEER are shown in Figure 2.

**Central Illustration:**
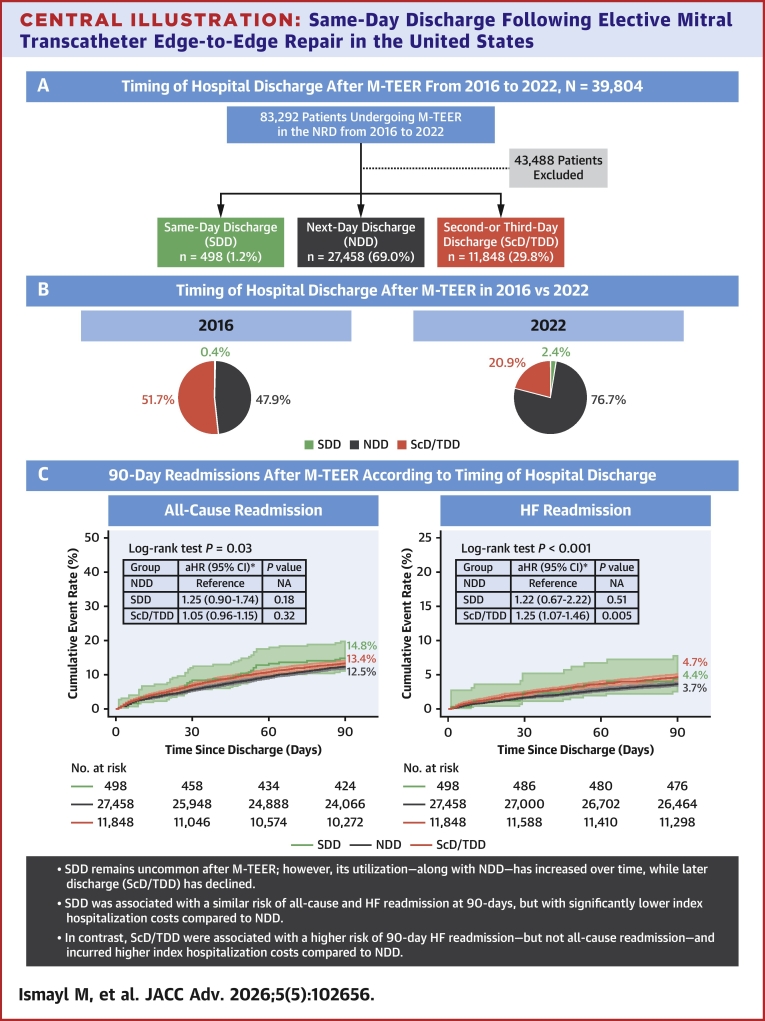
**Same-Day Discharge Following Elective Mitral Transcatheter Edge-to-Edge Repair in the United States** (A) Overall incidence of SDD, NDD, and ScD/TDD following M-TEER; (B) Incidence of SDD, NDD, and ScD/TDD following M-TEER in 2016 vs 2022; (C) 90-day readmissions after M-TEER according to timing of hospital discharge. ∗The multivariable regression model is adjusted for age, sex, primary payer, median income quartile by zip code, hospital location (urban/rural) and teaching status, number of hospital beds, Elixhauser and Charlson Comorbidity Index scores, relevant comorbidities, procedural complications, and discharge disposition (Supplemental Table 4). aHR = adjusted HR; HF = heart failure; M-TEER = mitral transcatheter edge-to-edge repair; NA = not applicable; NRD = Nationwide Readmissions Database.

### 30- and 90-day readmission

The estimated overall all-cause readmission rates were 6.7% (95% CI: 6.4% to 7.0%) at 30 days and 13.5% (95% CI: 13.1% to 13.9%) at 90 days. The most common cause for readmission was HF at both 30 days (2.1%; 95% CI: 1.9% to 2.3%) and 90 days (4.4%; 95% CI: 4.1% to 4.7%).

**Figure 3:**
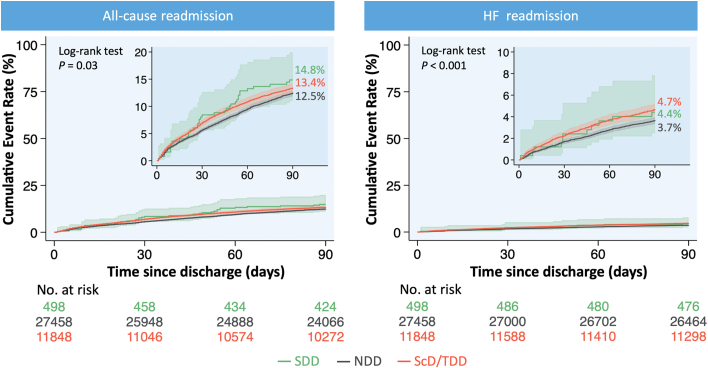
**Kaplan-Meier Curves of 90-Day Readmission Following Mitral Transcatheter Edge-to-Edge Repair Stratified by Timing of Hospital Discharge** HF = heart failure; other abbreviations as in Figures 1 and 2.

After adjustment for potential confounders using multivariable regression analysis, SDD was associated with a similar risk of all-cause and HF readmission at both 30 and 90 days compared to NDD. In contrast, later discharge (ScD/TDD) was associated with an increased risk of all-cause and HF readmission at 30 days, and an increased risk of HF readmission—but not all-cause readmission—at 90 days compared to NDD. Readmissions stratified by timing of hospital discharge are presented in Figure 3 and Table 2. These findings remained consistent in the sensitivity analyses excluding 1) hospitalizations involving any procedural complication (Supplemental Figure 1, Supplemental Table 6) and 2) those where patients were discharged to short-term hospitals or skilled nursing/intermediate care facilities (Supplemental Figure 2, Supplemental Table 7).

**Table 2:** Readmissions Stratified by Timing of Discharge

	SDD (n = 498)	NDD (n = 27,458)	ScD/TDD (n = 11,848)
30-day readmission			
All-cause readmission			
% (95% CI)	8.4 (5.6-12.6)	5.7 (5.3-6.1)	7.0 (6.4-7.7)
uHR (95% CI), *P* value	1.49 (0.96-2.29), 0.07	Ref.	1.25 (1.11-1.40), <0.001
aHR (95% CI), *P* value^a^	1.52 (0.98-2.38), 0.06	Ref.	1.18 (1.04-1.34), 0.01
Heart failure readmission			
% (95% CI)	2.4 (1.1-5.3)	1.7 (1.5-1.9)	2.3 (1.9-2.7)
uHR (95% CI), *P* value	1.41 (0.63-3.16), 0.41	Ref.	1.34 (1.08-1.65), 0.007
aHR (95% CI), *P* value^a^	1.46 (0.65-3.28), 0.36	Ref.	1.30 (1.04-1.63), 0.03
90-day readmission			
All-cause readmission			
% (95% CI)	14.8 (11-19.9)	12.5 (11.9-13.1)	13.4 (12.6-14.3)
uHR (95% CI), *P* value	1.20 (0.87-1.67), 0.26	Ref.	1.08 (0.99-1.18), 0.06
aHR (95% CI), *P* value^a^	1.25 (0.90-1.74), 0.18	Ref.	1.05 (0.96-1.15), 0.32
Heart failure readmission			
% (95% CI)	4.4 (2.5-7.8)	3.7 (3.4-4.0)	4.7 (4.2-5.3)
uHR (95% CI), *P* value	1.20 (0.66-2.18), 0.54	Ref.	1.28 (1.11-1.48), 0.001
aHR (95% CI), *P* value^a^	1.22 (0.67-2.22), 0.51	Ref.	1.25 (1.07-1.46), 0.005

aHR = adjusted HR; uHR = unadjusted HR; other abbreviations as in Table 1.

a The multivariable regression model is adjusted for age, sex, primary payer, median income quartile by zip code, hospital location (urban/rural) and teaching status, number of hospital beds, Elixhauser and Charlson Comorbidity Index scores, relevant comorbidities, procedural complications, and discharge disposition (Supplemental Table 4).

Propensity score matching yielded 498 pairs (n = 996). After matching, covariate balance was adequate, with absolute standardized mean differences <0.15 for all variables and <0.10 for most (Supplemental Figures 3 and 4). Similar to the multivariable regression analysis, SDD was associated with a similar risk of all-cause and HF readmission at both 30 and 90 days compared to NDD (Supplemental Table 8).

In a sensitivity analysis accounting for a plausible 1.2% 30-day out-of-hospital mortality based on the STS/ACC TVT Registry,^21^ readmission HRs were similar to the primary analysis (Supplemental Table 9), suggesting that our findings are unlikely to be materially affected by unobserved deaths.

### Total costs of index hospitalization and 90-day readmissions

Compared to NDD, SDD was associated with significantly lower index hospitalization costs (median [IQR]: $38,029 [$28,532–$61,038] vs $43,319 [$32,965–$65,161]; *P* = 0.03), whereas ScD/TDD were associated with significantly higher costs ($49,648 [$35,831–$75,816] vs $43,319 [$32,965–$65,161]; *P* < 0.001).

In contrast, 90-day readmission costs were similar between SDD and NDD ($14,348 [$5,392–$22,242] vs $12,411 [$5,289–$32,752]; *P* = 0.46), whereas ScD/TDD were associated with significantly lower readmission costs compared to NDD ($9,297 [$5,870–$14,590] vs $12,411 [$5,289–$32,752]; *P* < 0.001). Total costs of index hospitalization and 90-day readmissions stratified by timing of index hospital discharge are shown in Table 3.

**Table 3:** Total Index Hospitalization and 90-Day Readmission Costs Stratified by Timing of Discharge

	SDD (n = 498)	NDD (n = 27,458)	*P* Value^a^	ScD/TDD (n = 11,848)	*P* Value^b^
Index hospitalization ($)	38,029 (28,532-61,038)	43,319 (32,965-65,161)	0.03	49,648 (35,831-75,816)	<0.001
90-day readmission ($)	14,348 (5,392-22,242)	12,411 (5,289-32,752)	0.46	9,297 (5,870-14,590)	<0.001

Data presented as median (IQR).

Abbreviations as in Table 1.

a *P* values comparing total costs between SDD vs NDD using the Mann-Whitney *U* test.

b *P* values comparing total costs between ScD/TDD vs NDD using the Mann-Whitney *U* test.

### Predictors of SDD and 90-day all-cause readmission

In multivariable analysis, variables independently associated with lower odds of SDD following M-TEER included major bleeding complications (adjusted OR [aOR]: 0.12; 95% CI: 0.02 to 0.87), chronic pulmonary disease (aOR: 0.68; 95% CI: 0.51-0.89), renal failure (aOR: 0.34; 95% CI: 0.15-0.77), and nonhome discharge (aOR: 0.17; 95% CI: 0.10-0.31).

**Figure 4:**
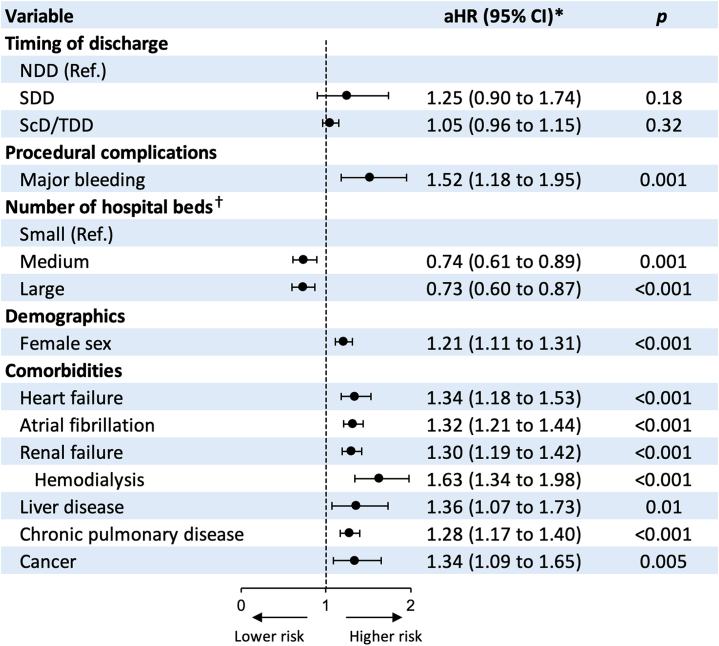
**Forest Plot for Independent Predictors of 90-Day All-Cause Readmission Following Mitral Transcatheter Edge-To-Edge Repair** ∗The multivariable regression model is adjusted for age, sex, primary payer, median income quartile by zip code, hospital location (urban/rural) and teaching status, number of hospital beds, Elixhauser and Charlson Comorbidity Index scores, relevant comorbidities, procedural complications, and discharge disposition (Supplemental Table 4). †Bed-size categories are based on inpatient beds and are specific to the hospital’s location and teaching status. aHR = adjusted HR; other abbreviations as in Figures 1 and 2.

In multivariable analysis, variables independently associated with higher 90-day all-cause readmission following M-TEER hospitalization included major bleeding complications, female sex, and a history of HF, atrial fibrillation, renal failure, hemodialysis dependence, liver disease, chronic pulmonary disease, or cancer. Conversely, index admission to hospitals with higher bed capacity was independently associated with a lower risk of 90-day readmission. Variables independently associated with 90-day all-cause readmission are presented in Figure 4.

## Discussion

In this nationally representative analysis of readmissions following M-TEER hospitalizations, we identified several key findings regarding the timing of hospital discharge (Central Illustration): 1) SDD remains uncommon after M-TEER; however, its utilization—along with NDD—has significantly increased over time, whereas later discharge (ScD/TDD) has declined; 2) compared with NDD, SDD was associated with a similar risk of 90-day all-cause and HF readmission, but with significantly lower index hospitalization costs; 3) in contrast, ScD/TDD was associated with a higher risk of 90-day HF readmission—but not all-cause readmission—and incurred higher index hospitalization costs compared to NDD; 4) major bleeding complications, history of chronic pulmonary disease or renal failure, and nonhome discharge were associated with lower likelihood of SDD; and 5) independent predictors of higher 90-day all-cause readmission following M-TEER included major bleeding complications, female sex, and a history of HF, atrial fibrillation, renal failure, hemodialysis dependence, liver disease, chronic pulmonary disease, or cancer.

### Temporal trends in SDD following M-TEER

Since the mid-2010s, the evolution of structural heart interventions has been accompanied by a paradigm shift favoring early discharge protocols, aimed at increasing patient throughput and reducing health care costs.^3^ The present study demonstrates a clear shift in hospital discharge practices following M-TEER from 2016 to 2022. Although SDD remains infrequent, its use has increased 6-fold during this time period, reflecting a growing interest in early discharge strategies among centers performing M-TEER. This upward trend mirrors the trajectory of SDD observed in other structural interventions, such as TAVR and LAAO, where early discharge protocols have evolved from niche practices to mainstream strategies in selected patient populations.^4^^,^^7^

This increase in SDD frequency likely reflects increased operator experience, improved procedural techniques, and enhanced postprocedural monitoring. In addition, institutional efforts to reduce hospital LOS for elective procedures—particularly during and after the COVID-19 pandemic—may have further accelerated this trend. Although prior studies have demonstrated that M-TEER can be safely performed under conscious or deep sedation,^11^^,^^28^ general anesthesia remains the standard approach at most institutions. This preference is largely driven by the requirement for transesophageal echocardiography guidance and the procedural stability afforded by low tidal volume ventilation or brief apnea during leaflet grasping. Nevertheless, immediate postprocedural extubation in the procedure suite is often feasible^10^, enabling adequate postanesthetic monitoring before considering SDD. In addition, the emergence of 3-dimensional intracardiac echocardiography as a potential alternative to transesophageal echocardiography for imaging guidance in structural heart interventions, including M-TEER, may further support the adoption of SDD.^29^ Furthermore, given direct access to the left atrium during the procedure, operators are well-positioned to assess intravascular volume status as reflected by left atrial pressure and to determine whether extended hospitalization is necessary for diuresis or optimization of HF guideline-directed medical therapy.

As operators increasingly adopt SDD—particularly at high-volume, resource-optimized centers—its broader implementation in the M-TEER population warrants thoughtful evaluation. Careful patient selection is essential, as clinical factors such as major bleeding complications, chronic pulmonary disease, and renal failure were independently associated with both reduced likelihood of SDD and increased risk of 90-day all-cause readmission. These findings highlight the importance of individualized discharge planning to ensure both safety and optimal postprocedural outcomes.

### 30- and 90-day readmission

A primary concern with early discharge strategies is the potential for increased readmission due to premature transition to outpatient care. However, the present results provide reassurance: SDD was associated with similar 30- and 90-day all-cause and HF readmission rates compared with NDD. These findings align with prior studies of early discharge in TAVR and LAAO, which have shown that appropriately selected patients do not experience increased adverse events after early discharge.^4^, ^5^, ^6^, ^7^, ^8^ Furthermore, the absolute rates of HF readmission in the present study were low and improved over time when compared with earlier data from the STS/ACC TVT Registry, which reported higher HF readmission rates among patients undergoing M-TEER between 2013 and 2015.^30^

In contrast, later discharge (ScD/TDD) was associated with a significantly higher risk of 30-day all-cause and HF readmission, as well as increased 90-day HF readmission, compared to NDD. These findings likely reflect the greater baseline risk and higher burden of in-hospital complications among patients requiring prolonged hospitalization. Prior studies have demonstrated that extended hospital LOS often correlates with underlying clinical complexity or periprocedural complications, which independently increase readmission risk.^6^^,^^31^^,^^32^ In our study, patients in the ScD/TDD group exhibited higher rates of coagulopathy and periprocedural complications, including major bleeding, and were more frequently discharged to skilled nursing or intermediate care facilities. Major bleeding, along with chronic organ dysfunction and female sex, were identified as independent predictors of 90-day readmission in the present study, highlighting the complex interplay of clinical and demographic factors associated with postdischarge vulnerability in this population. These findings align with prior studies in both M-TEER and TAVR populations, reinforcing the importance of an individualized approach to discharge planning and transitional care for high-risk patients.^6^^,^^31^^,^^32^

### Index hospitalization costs

Hospital LOS is a key determinant of cost in structural heart interventions.^5^^,^^6^^,^^8^ SDD was associated with significantly lower index hospitalization costs compared to NDD, whereas ScD/TDD incurred the highest costs. These findings are consistent with similar cost analyses in the TAVR and LAAO literature, where early discharge reduced resource utilization substantially without adversely affecting clinical outcomes.^5^^,^^6^^,^^8^ With the continued expansion of M-TEER indications and volumes, cost-effectiveness will remain a critical metric for health care systems and payers. The ability to discharge patients safely on the day of the procedure not only reduces direct hospitalization costs but also improves bed turnover and resource allocation. Given that SDD did not increase readmission risk in our cohort, these findings suggest an opportunity for cost containment and care optimization through more widespread implementation of early discharge protocols in appropriately selected M-TEER patients.

### Future directions

Although the present study supports the feasibility and safety of SDD in carefully selected M-TEER patients, further prospective validation is needed. In addition, developing standardized criteria for SDD eligibility—potentially incorporating frailty scores, access site characteristics, procedural success, degree of residual MR, and early recovery indicators—will be essential to ensure patient safety. Furthermore, incorporating patient-reported outcomes, caregiver burden, and satisfaction into future studies may provide a more comprehensive assessment of the benefits and limitations of SDD, facilitating patient-centered care planning. Finally, leveraging digital health platforms for postdischarge monitoring may also facilitate safe early discharge while maintaining quality of care.^33^ Remote blood pressure, heart rate and rhythm, and symptom tracking platforms may allow clinicians to identify postprocedural complications early, reducing the need for prolonged hospitalization for monitoring.^33^

### Study Limitations and strengths

This study has several important limitations to acknowledge. First, in a retrospective NRD study using administrative claims codes, incorrect coding could lead to inaccurate data. Second, because SDD was identified using a LOS of 0 days in the NRD, some overnight hospitalizations not recorded as a separate day may have been misclassified, representing an inherent limitation of administrative data. Third, the retrospective nature of the study makes it subject to inherent selection bias, and prospective studies are needed to confirm the present findings. Fourth, despite multivariable regression analyses, propensity score matching, and sensitivity analyses to adjust for potential confounders, there may remain unmeasured confounders that could affect the findings of this study. Fifth, detailed patient-level factors, including frailty, cognitive status, and social support, which are key determinants for safe SDD, as well as procedural characteristics, such as echocardiographic and computed tomographic data, access site, device type (eg, MitraClip or the PASCAL system), number of devices deployed, and periprocedural medications, are unavailable in the NRD, which can lead to unmeasured bias. Sixth, validated risk scores such as the Society of Thoracic Surgeons score are not captured by the NRD, limiting patient risk assessment. Seventh, the low proportion of SDD cases (1.2%) suggests that this approach was reserved for highly selected patients at experienced, high-volume centers, and may not be broadly generalizable to all centers. Eighth, given the lack of data on out-of-hospital deaths, patients who died at home within 90 days were counted as patients without a readmission within 90 days; this limitation precludes formal competing risk analyses.^12^ However, in a sensitivity analysis assuming a plausible 30-day out-of-hospital mortality rate of 1.2%, results remained consistent with the primary analysis. Finally, because the NRD does not allow longitudinal tracking of patients beyond a single calendar year, we were unable to report a median and IQR of follow-up time; instead, follow-up was limited to a maximum of 90 days after discharge. Studies exploring the long-term safety of SDD following M-TEER hospitalizations are still needed.

Despite these limitations, this study adds meaningfully to the literature by describing the feasibility and safety of SDD following M-TEER in the United States. The NRD is well validated for outcomes studies like this one and undergoes serial data accuracy checks and quality control. In addition, NRD data are geographically diverse, including a nationally representative sample of centers and operators, and hence reliably reflect real-world practice and outcomes.

## Conclusions

In this large, nationally representative observational study, SDD following M-TEER was uncommon but associated with a similar risk of 90-day readmission and lower index hospitalization costs compared to NDD. These findings support the feasibility of SDD following M-TEER in carefully selected patients, highlighting the need for prospective studies to further evaluate its safety and to refine criteria for optimal patient selection.

Perspectives **COMPETENCY IN MEDICAL KNOWLEDGE:** SDD following structural heart interventions has garnered increasing attention in recent years. SDD remains uncommon after M-TEER; however, its utilization has increased 6-fold from 2016 to 2022. SDD after M-TEER is associated with a similar risk of 90-day readmission and lower index hospitalization costs compared to NDD. These findings support the feasibility and safety of SDD in carefully selected M-TEER patients.

**TRANSLATIONAL OUTLOOK:** Further research is warranted to identify the optimal patient selection criteria for SDD after M-TEER.

## Funding support and author disclosures

This work was supported by the Department of Cardiovascular Medicine at Mayo Clinic in Rochester, Minnesota. Dr Goldsweig has done consulting for 10.13039/100004320Philips, 10.13039/100000046Abbott, Occlutech, and Conformal Medical; and has done speaking for 10.13039/100004320Philips and 10.13039/100008497Boston Scientific. Dr Rihal has received research funding from 10.13039/100006520Edwards Lifesciences. Dr Guerrero has received institutional research grant support from 10.13039/100006520Edwards Lifesciences. All other authors have reported that they have no relationships relevant to the contents of this paper to disclose.
