# Supplementary material for: Feasibility and Safety of Same-Day Discharge Following Elective Mitral Transcatheter Edge-to-Edge Repair
Source: JACC Adv. 2026 Apr 25;5(5):102656. doi: 10.1016/j.jacadv.2026.102656 (PMC13221869; doi:10.1016/j.jacadv.2026.102656)

**Supplementary Material**

**Supplemental Table 1.** Summary of NRD states, hospitals, and inpatient stays (adopted from <https://hcup-us.ahrq.gov/db/nation/nrd/NRDIntroduction2022.pdf>)

| **Year** | **States** | **Number of States for Discharges (Age ≥1 Year)** | **Number of Hospitals** | **Number of Discharges in the NRD** | |
| --- | --- | --- | --- | --- | --- |
|  |  |  |  | **Unweighted** | **Weighted** |
| 2022 | AK, AR, CA, CO, DE, FL, GA, HI, IA, IN, LA, MA, MD, ME, MO, MS, NE, NM, NY, OR, PA, SC, SD, TN, UT, VA, VT, WA, WI, WY | 30 | 2,505 | 16,517,253 | 32,886,431 |
| 2021 | AK, AR, CA, CO, DE, FL, GA, HI, IA, IN, LA, MA, MD, ME, MO, MS, NE, NM, NY, OR, PA, SC, SD, TN, UT, VA, VT, WA, WI, WY | 30 | 2,505 | 16,805,508 | 33,420,845 |
| 2020 | AK, AR, CA, CO, DE, FL, GA, HI, IA, IN, LA, MA, MD, ME, MO, MS, NE, NM, NV, NY, OR, PA, SC, SD, TN, UT, VA, VT, WA, WI, WY | 31 | 2,539 | 16,692,694 | 32,336,475 |
| 2019 | AK, AR, CA, CO, DE, FL, GA, HI, IA, IN, LA, MA, MD, MO, MS, NE, NM, NV, NY, OR, PA, SC, SD, TN, UT, VA, VT, WA, WI, WY | 30 | 2,507 | 18,132,856 | 35,399,480 |
| 2018 | AK, AR, CA, DE, FL, GA, IA, IN, LA, MA, MD, MO, MS, NE, NM, NV, NY, OR, PA, SC, SD, TN, UT, VA, VT, WA, WI, WY | 28 | 2,430 | 17,686,511 | 35,460,557 |
| 2017 | AK, AR, CA, DE, FL, GA, IA, IN, LA, MA, MD, MO, MS, NE, NM, NV, NY, OR, PA, SC, SD, TN, UT, VA, VT, WA, WI, WY | 28 | 2,454 | 17,978,754 | 35,790,513 |
| 2016 | AK, AR, CA, FL, GA, HI, IA, LA, MA, MD, MO, MS, NE, NM, NV, NY, OR, PA, SC, SD, TN, UT, VA, VT, WA, WI, WY | 27 | 2,355 | 17,197,683 | 35,660,906 |

# Supplemental Table 2. Strengthening the Reporting of Observational Studies in Epidemiology (STROBE) guidelines to report the study findings

STROBE Statement—Checklist of items that should be included in reports of ***cross-sectional studies***

|  | **Item No** | **Recommendation** | **Page No** |
| --- | --- | --- | --- |
| **Title and abstract** | 1 | (*a*) Indicate the study’s design with a commonly used term in the title or the abstract | 3 |
|  |  | (*b*) Provide in the abstract an informative and balanced summary of what was done and what was found | 3,4 |
| **Introduction** | | | |
| Background/rationale | 2 | Explain the scientific background and rationale for the investigation being reported | 6 |
| Objectives | 3 | State specific objectives, including any prespecified hypotheses | 6 |
| **Methods** | | | |
| Study design | 4 | Present key elements of study design early in the paper | 7,8 |
| Setting | 5 | Describe the setting, locations, and relevant dates, including periods of recruitment, exposure, follow-up, and data collection | 7,8 |
| Participants | 6 | (*a*) Give the eligibility criteria, and the sources and methods of selection of participants | 7,8 |
| Variables | 7 | Clearly define all outcomes, exposures, predictors, potential confounders, and effect modifiers. Give diagnostic criteria, if applicable | 8,9 |
| Data sources/ measurement | 8* | For each variable of interest, give sources of data and details of methods of assessment (measurement). Describe comparability of assessment methods if there is more than one group | 6-8 |
| Bias | 9 | Describe any efforts to address potential sources of bias | 10,11 |
| Study size | 10 | Explain how the study size was arrived at | 7-9 |
| Quantitative variables | 11 | Explain how quantitative variables were handled in the analyses. If applicable, describe which groupings were chosen and why | 7-10 |
| Statistical methods | 12 | (*a*) Describe all statistical methods, including those used to control for confounding | 10,11 |
|  |  | (*b*) Describe any methods used to examine subgroups and interactions | N/A |
|  |  | (*c*) Explain how missing data were addressed | 11 |
|  |  | (*d*) If applicable, describe analytical methods taking account of sampling strategy | 10,11 |
|  |  | (*e*) Describe any sensitivity analyses | N/A |
| **Results** | | | |
| Participants | 13* | (a) Report numbers of individuals at each stage of study—eg numbers potentially eligible, examined for eligibility, confirmed eligible, included in the study, completing follow-up, and analysed | 11 |
|  |  | (b) Give reasons for non-participation at each stage | 11 |
|  |  | (c) Consider use of a flow diagram | 11 |
| Descriptive data | 14* | (a) Give characteristics of study participants (eg demographic, clinical, social) and information on exposures and potential confounders | 12,13 |
|  |  | (b) Indicate number of participants with missing data for each variable of interest | 11 |
| Outcome data | 15* | Report numbers of outcome events or summary measures | 12-14 |
| Main results | 16 | (*a*) Give unadjusted estimates and, if applicable, confounder-adjusted estimates and their precision (eg, 95% confidence interval). Make clear which confounders were adjusted for and why they were included | 12-14 |
|  |  | (*b*) Report category boundaries when continuous variables were categorized | N/A |
|  |  | (*c*) If relevant, consider translating estimates of relative risk into absolute risk for a meaningful time period | N/A |
| Other analyses | 17 | Report other analyses done—eg analyses of subgroups and interactions, and sensitivity analyses | N/A |
| **Discussion** | | | |
| Key results | 18 | Summarise key results with reference to study objectives | 14 |
| Limitations | 19 | Discuss limitations of the study, taking into account sources of potential bias or imprecision. Discuss both direction and magnitude of any potential bias | 17,18 |
| Interpretation | 20 | Give a cautious overall interpretation of results considering objectives, limitations, multiplicity of analyses, results from similar studies, and other relevant evidence | 14-17 |
| Generalisability | 21 | Discuss the generalisability (external validity) of the study results | 14-18 |
| **Other information** | | | |
| Funding | 22 | Give the source of funding and the role of the funders for the present study and, if applicable, for the original study on which the present article is based | 1 |

*Give information separately for exposed and unexposed groups.

**Note:** An Explanation and Elaboration article discusses each checklist item and gives methodological background and published examples of transparent reporting. The STROBE checklist is best used in conjunction with this article (freely available on the Web sites of PLoS Medicine at http://www.plosmedicine.org/, Annals of Internal Medicine at http://www.annals.org/, and Epidemiology at http://www.epidem.com/). Information on the STROBE Initiative is available at www.strobe-statement.org.

**Supplemental Table 3.** ICD-10 diagnosis (CM) and procedure (PCS) codes

| **Inclusion** | **ICD-10 PCS codes** |
| --- | --- |
| M-TEER | 02UG3JH, 02QG3ZE, 02QG3ZZ, 02UG3JZ, 02UG37E, 02UG37Z, 02UG38E, 02UG38Z, 02UG3JE, 02UG3KE, 02UG3KZ, 02UG47E, 02UG47Z, 02UG48E, 02UG48Z, 02UG4JE, 02UG4JZ, 02UG4KE, 02UG4KZ |

| **Baseline characteristics** | **ICD-10 CM codes** |
| --- | --- |
| Diabetes mellitus | E10.0, E10.1, E10.9, E11.0, E11.1, E11.9, E12.0, E12.1, E12.9, E13.0, E13.1, E13.9, E14.0, E14.1, E14.9, E10.2-E10.8, E11.2-E11.8, E12.2-E12.8, E13.2-E13.8, E14.2-E14.8 |
| Hypertension | I10.x, I11.x-I13.x, I15.x |
| Dyslipidemia | E78.x |
| Nicotine/tobacco use | F17.x, Z72.0, Z87.891 |
| Alcohol abuse | F10, E52, G62.1, I42.6, K29.2, K70.0, K70.3, K70.9, T51.x, Z50.2, Z71.4, Z72.1 |
| Drug abuse | F11.x-F16.x, F18.x, F19.x, Z71.5. Z72.2 |
| Obesity | E66.x |
| Coronary artery disease | I25.x |
| Peripheral artery disease | I70.x, I71.x, I73.1, I73.8, I73.9, I77.1, I79.0, I79.2, K55.1, K55.8, K55.9, Z95.8, Z95.9 |
| Atrial fibrillation/flutter | I48.x |
| Congestive heart failure | I09.9, I11.0, I13.0, I13.2, I25.5, I42.0, 142.5-I42.9, I43.x, I50.x, P29.0 |
| Renal failure | I12.0, I13.1, N18.x, N19.x, N25.0, Z49.0-Z49.2, Z94.0, Z199.2 |
| Dialysis dependent | Z99.2 |
| Liver disease | B18.x, I85.x, I86.4, I98.2, K70.x, K71.1, K71.3-K71.5, K71.7, K72.x-K74.x, K76.0, K76.2-K76.9. Z94.4 |
| Chronic pulmonary disease | I27.8, 127.9, J40.x-J47.x, J60.x-J67.x, J68.4, J70.1, J70.3 |
| Obstructive sleep apnea | G47.33 |
| Coagulopathy | D65-D68.x, D69.1, D69.3-D69.6 |
| Cancer | C0x.x, C1x.x, C2x.x, C30.x, C31.x, C32.x, C33.x, C34.x, C37.x, C38.x, C39.x, C40.x, C41.x, C43.x, C45.x, C46.x, C47.x, C48.x, C49.x, C50, C51-58.x, C60-63.x, C76.x, C80.1, C81.x, C82.x, C83.x, C84.x, C85.x, C88.x, C9x.x |
| Malnutrition | E43, E44.x, E45, E46 |
| Dementia | F01.x, F02.x, F03.x, F04, F05, F06.1, F06.8, G13.2, G13.8, G30.x, G31.0x, G31.1, G31.2, G91.4, G94, R41.81, R54 |
| Depression | F20.4, F31.3-F31.5, F32.x, F33.x, F34.1, F41.2, F43.2 |
| **Previous history** | |
| Myocardial infarction | I25.2 |
| Stroke/TIA | Z86.73 |
| Cardiac arrest | Z86.74 |
| PCI | Z98.61, Z95.5 |
| CABG | Z95.1 |
| ICD | Z95.810 |
| PPM | Z95.0 |

| **Procedural complications** | **ICD-10 CM/PCS codes** |
| --- | --- |
| Stroke | I60.x, I61.x, I62.x, I63.x, I67.81, I67.82, G45.x, G46.x, H34.0x, H34.1x, H34.2x, I97.820, I97.810 |
| Major bleeding | I97.610, I97.618, I97.620, I97.410, I97.418, I97.42, T82.837 |
| Blood transfusion | 30233H0, 30233H1, 30233N0, 30233N1, 30233P0, 30233P1, 30230H0, 30230H1, 30230N0, 30230N1, 30230P0, 30230P1, 30243H0, 30243H1, 30243N0, 30243N1, 30243P0, 30243P1, 30240H0, 30240H1, 30240N0, 30240N1, 30240P0, 30240P1 |
| Vascular complications | I77.0, I72.x, L76.32, L76.02, I97.630, I97.638, K66.1, I26.92, I26.93, I26.94, I26.99, I26.02, I26.09, I82.4x, T81.718A, T81.719A |
| Acute kidney injury | N17.x, N99.0 |

*Abbreviations.* CABG=coronary artery bypass grafting; ICD=implantable cardioverter-defibrillator; M-TEER=mitral transcatheter edge-to-edge repair; PCI=percutaneous coronary intervention; PPM=permanent pacemaker; TIA=transient ischemic attack.

**Supplemental Table 4.** Variables used in the multivariable regression analysis to compute adjusted readmissions as well as independent predictors of SDD and 90-all-cause readmission

| **Demographic characteristics** |
| --- |
| Age |
| Biological sex |
| Primary payer |
| Income quartile |
| **Hospital characteristics** |
| Location/teaching status |
| Bed size |
| **Comorbidities** |
| Elixhauser comorbidity index |
| Charlson comorbidity index |
| Diabetes mellitus |
| Hypertension |
| Dyslipidemia |
| Nicotine/tobacco use |
| Alcohol abuse |
| Drug abuse |
| Obesity |
| Coronary artery disease |
| Peripheral artery disease |
| Atrial fibrillation/flutter |
| Congestive heart failure |
| Renal failure |
| Dialysis dependent |
| Liver disease |
| Chronic pulmonary disease |
| Obstructive sleep apnea |
| Coagulopathy |
| Cancer |
| Malnutrition |
| Dementia |
| Depression |
| **Previous** **history** |
| Myocardial infarction |
| Stroke/TIA |
| Cardiac arrest |
| PCI |
| CABG |
| ICD |
| PPM |
| **Procedural complications** (excluded from sensitivity analysis) |
| Stroke |
| Major bleeding |
| Need for blood transfusion |
| Vascular complications |
| Acute kidney injury |
| **Discharge disposition** (excluded from sensitivity analysis) |

*Abbreviations.* CABG=coronary artery bypass grafting; ICD=implantable cardioverter-defibrillator; PCI=percutaneous coronary intervention; PPM=permanent pacemaker; TIA=transient ischemic attack.

| **Demographic characteristics** |
| --- |
| Age |
| Biological sex |
| Primary payer |
| Income quartile |
| **Hospital characteristics** |
| Location/teaching status |
| Bed size |
| **Comorbidities** |
| Elixhauser comorbidity index |
| Charlson comorbidity index |
| Diabetes mellitus |
| Hypertension |
| Dyslipidemia |
| Alcohol abuse |
| Obesity |
| Peripheral artery disease |
| Congestive heart failure |
| Renal failure |
| Dialysis dependent |
| Liver disease |
| Chronic pulmonary disease |
| Obstructive sleep apnea |
| Coagulopathy |
| Cancer |
| Dementia |
| Depression |
| **Previous** **history** |
| Myocardial infarction |
| Stroke/TIA |
| Cardiac arrest |
| PCI |
| CABG |
| ICD |
| **Procedural complications** |
| Stroke |
| Major bleeding |
| Need for blood transfusion |
| Vascular complications |
| Acute kidney injury |

**Supplemental Table 5.** Variables used in propensity score matching to compute adjusted readmissions

*Abbreviations.* CABG=coronary artery bypass grafting; ICD=implantable cardioverter-defibrillator; PCI=percutaneous coronary intervention; TIA=transient ischemic attack.

**Supplemental Table 6.** Sensitivity analysis of readmissions stratified by timing of discharge after excluding patients with procedural complications during index hospitalization

|  | **SDD**  (*n*=488) | **NDD**  (*n*=26,480) | **ScD/TDD**  (*n*=10,258) |
| --- | --- | --- | --- |
| **30-day readmission** |  |  |  |
| **All-cause readmission** | 8.2 | 5.6 | 6.5 |
| uHR (95% CI) | 1.47 (0.94-2.29) | Ref. | 1.17 (1.03-1.33) |
| aHR (95% CI)^a^ | 1.50 (0.95-2.37) | Ref. | 1.19 (1.04-1.35) |
| **Heart failure readmission** | 2.5 | 1.7 | 2.1 |
| uHR (95% CI) | 1.45 (0.65-3.26) | Ref. | 1.25 (0.99-1.56) |
| aHR (95% CI)^a^ | 1.57 (0.70-3.54) | Ref. | 1.30 (1.03-1.64) |
| **90-day readmission** |  |  |  |
| **All-cause readmission** | 14.3 | 12.4 | 12.8 |
| uHR (95% CI) | 1.16 (0.84-1.63) | Ref. | 1.03 (0.94-1.13) |
| aHR (95% CI)^a^ | 1.22 (0.87-1.71) | Ref. | 1.05 (0.96-1.15) |
| **Heart failure readmission** | 4.1 | 3.7 | 4.5 |
| uHR (95% CI) | 1.12 (0.60-2.10) | Ref. | 1.22 (1.04-1.43) |
| aHR (95% CI)^a^ | 1.16 (0.62-2.18) | Ref. | 1.26 (1.08-1.48) |

Data presented as % or HR (95% CI).

^a^The multivariable regression model is adjusted for age, sex, primary payer, median income quartile by ZIP code, hospital location (urban/rural) and teaching status, number of hospital beds, Elixhauser and Charlson comorbidity index scores, relevant comorbidities, and discharge disposition (*Supplemental* *Table 4*).

*Abbreviations*. aHR=adjusted hazard ratio; CI=confidence interval; NDD=Next-Day Discharge; SDD=Same-Day Discharge; ScD/TDD=Second- or Third-Day Discharge; uHR=unadjusted hazard ratio.

**Supplemental Table 7.** Sensitivity analysis of readmissions stratified by timing of discharge after excluding patients who were transferred to a short-term hospital or a skilled nursing/intermediate care facility

|  | **SDD**  (*n*=496) | **NDD**  (*n*=27,238) | **ScD/TDD**  (*n*=11,469) |
| --- | --- | --- | --- |
| **30-day readmission** |  |  |  |
| **All-cause readmission** | 8.4 | 5.6 | 6.9 |
| uHR (95% CI) | 1.51 (0.98-2.32) | Ref. | 1.23 (1.09-1.39) |
| aHR (95% CI)^a^ | 1.52 (0.97-2.37) | Ref. | 1.21 (1.06-1.37) |
| **Heart failure readmission** | 2.4 | 1.7 | 2.2 |
| uHR (95% CI) | 1.43 (0.64-3.21) | Ref. | 1.34 (1.08-1.65) |
| aHR (95% CI)^a^ | 1.45 (0.65-3.28) | Ref. | 1.32 (1.06-1.65) |
| **90-day readmission** |  |  |  |
| **All-cause readmission** | 14.9 | 12.5 | 13.3 |
| uHR (95% CI) | 1.22 (0.88-1.68) | Ref. | 1.07 (0.99-1.17) |
| aHR (95% CI)^a^ | 1.25 (0.90-1.74) | Ref. | 1.06 (0.97-1.16) |
| **Heart failure readmission** | 4.4 | 3.6 | 4.7 |
| uHR (95% CI) | 1.22 (0.67-2.21) | Ref. | 1.29 (1.11-1.49) |
| aHR (95% CI)^a^ | 1.22 (0.67-2.22) | Ref. | 1.29 (1.11-1.51) |

Data presented as % or HR (95% CI).

^a^The multivariable regression model is adjusted for age, sex, primary payer, median income quartile by ZIP code, hospital location (urban/rural) and teaching status, number of hospital beds, Elixhauser and Charlson comorbidity index scores, relevant comorbidities, and procedural complications (*Supplemental* *Table 4*).

*Abbreviations*. aHR=adjusted hazard ratio; CI=confidence interval; NDD=Next-Day Discharge; SDD=Same-Day Discharge; ScD/TDD=Second- or Third-Day Discharge; uHR=unadjusted hazard ratio.

**Supplemental Table 8.** Readmissions stratified by SDD vs. NDD after propensity score matching

|  | **SDD**  (*n*=498) | **NDD**  (*n*=498) | ***p* value** |
| --- | --- | --- | --- |
| **30-day readmission** |  |  |  |
| **All-cause readmission** | 7.4 | 5.6 | 0.49 |
| aHR (95% CI)^a^ | 1.33 (0.68-2.60) | Ref. | 0.40 |
| **Heart failure readmission** | 2.3 | 2.6 | 0.90 |
| aHR (95% CI)^a^ | 0.86 (0.29-2.55) | Ref. | 0.78 |
| **90-day readmission** |  |  |  |
| **All-cause readmission** | 11.0 | 13.9 | 0.28 |
| aHR (95% CI)^a^ | 0.77 (0.49-1.21) | Ref. | 0.25 |
| **Heart failure readmission** | 4.1 | 4.1 | >0.99 |
| aHR (95% CI)^a^ | 1.00 (0.43-2.31) | Ref. | >0.99 |

Data presented as % or HR (95% CI).

^a^Propensity score matching was performed based on age, sex, primary payer, median income quartile by ZIP code, hospital location (urban/rural) and teaching status, number of hospital beds, Elixhauser and Charlson comorbidity index scores, relevant comorbidities, and procedural complications (*Supplemental* *Table 5*).

*Abbreviations*. aHR=adjusted hazard ratio; CI=confidence interval; NDD=Next-Day Discharge; SDD=Same-Day Discharge.

**Supplemental Table 9.** Sensitivity analysis of 30-day readmissions stratified by timing of discharge after accounting for plausible 1.2% out-of-hospital mortality

|  | **SDD**  (*n*=498) | **NDD**  (*n*=27,458) | **ScD/TDD**  (*n*=11,848) |
| --- | --- | --- | --- |
| **30-day readmission** |  |  |  |
| **All-cause readmission** | 9.2 | 7.7 | 8.2 |
| uHR (95% CI) | 1.33 (0.86-2.05) | Ref. | 1.16 (1.03-1.31) |
| aHR (95% CI)^a^ | 1.50 (0.96-2.34) | Ref. | 1.13 (1.01-1.28) |
| **Heart failure readmission** | 3.4 | 3.8 | 4.2 |
| uHR (95% CI) | 1.33 (0.59-2.99) | Ref. | 1.30 (1.05-1.61) |
| aHR (95% CI)^a^ | 1.46 (0.65-3.28) | Ref. | 1.27 (1.01-1.59) |

Data presented as % or HR (95% CI).

^a^The multivariable regression model is adjusted for age, sex, primary payer, median income quartile by ZIP code, hospital location (urban/rural) and teaching status, number of hospital beds, Elixhauser and Charlson comorbidity index scores, relevant comorbidities, procedural complications, and discharge disposition (*Supplemental* *Table 4*).

*Abbreviations*. aHR=adjusted hazard ratio; CI=confidence interval; NDD=Next-Day Discharge; SDD=Same-Day Discharge; ScD/TDD=Second- or Third-Day Discharge; uHR=unadjusted hazard ratio.

**Supplemental Figure 1.** Kaplan-Meier curves of 90-day readmission stratified by timing of hospital discharge after excluding patients with procedural complications during index hospitalization. HF=heart failure; NDD=Next-Day Discharge; ScD/TDD=Second- or Third-Day Discharge; SDD=Same-Day Discharge.


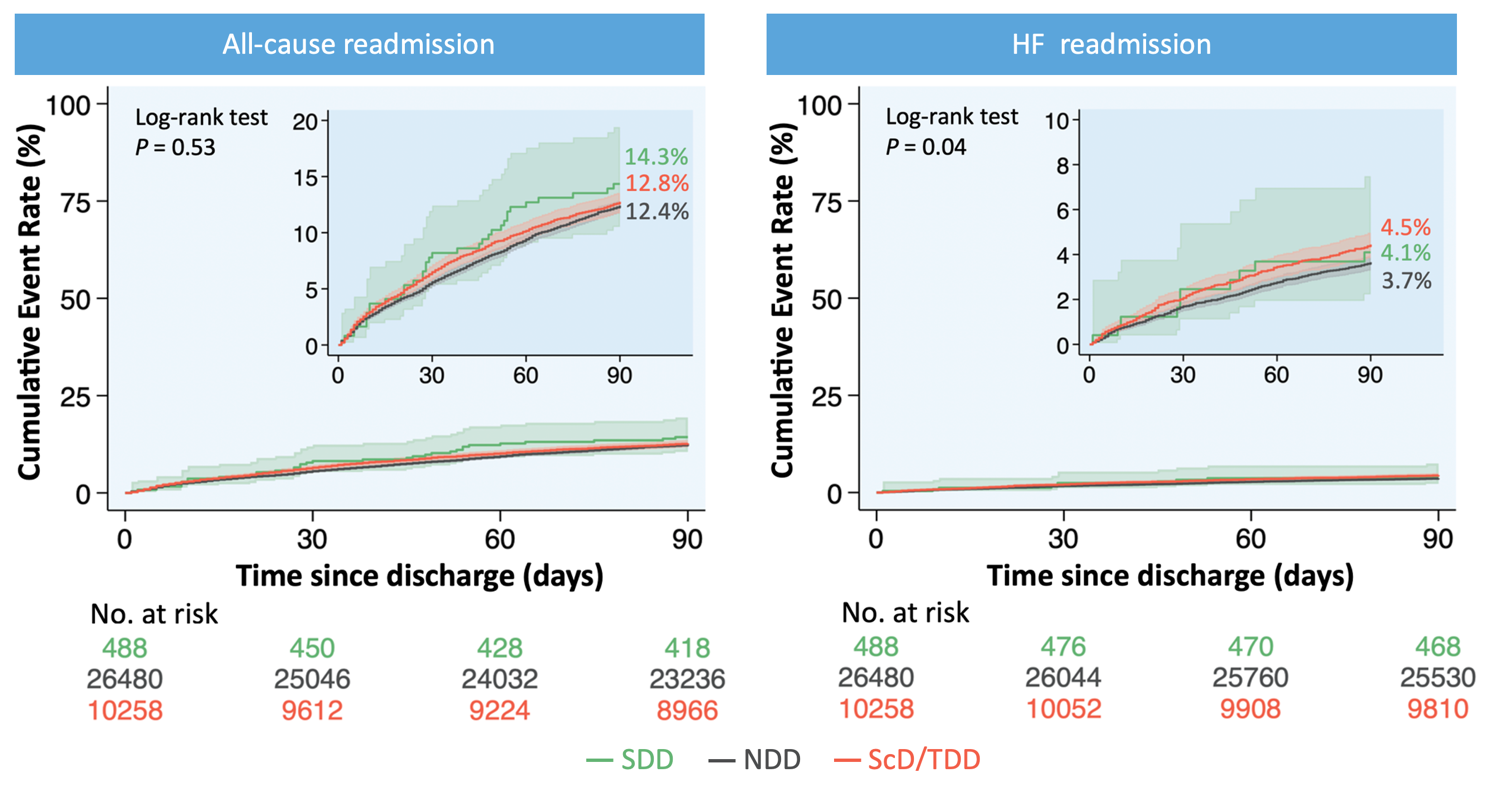


**Supplemental Figure 2.** Kaplan-Meier curves of 90-day readmission stratified by timing of hospital discharge after excluding patients who were transferred to a short-term hospital or a skilled nursing/intermediate care facility. HF=heart failure; NDD=Next-Day Discharge; ScD/TDD=Second- or Third-Day Discharge; SDD=Same-Day Discharge.


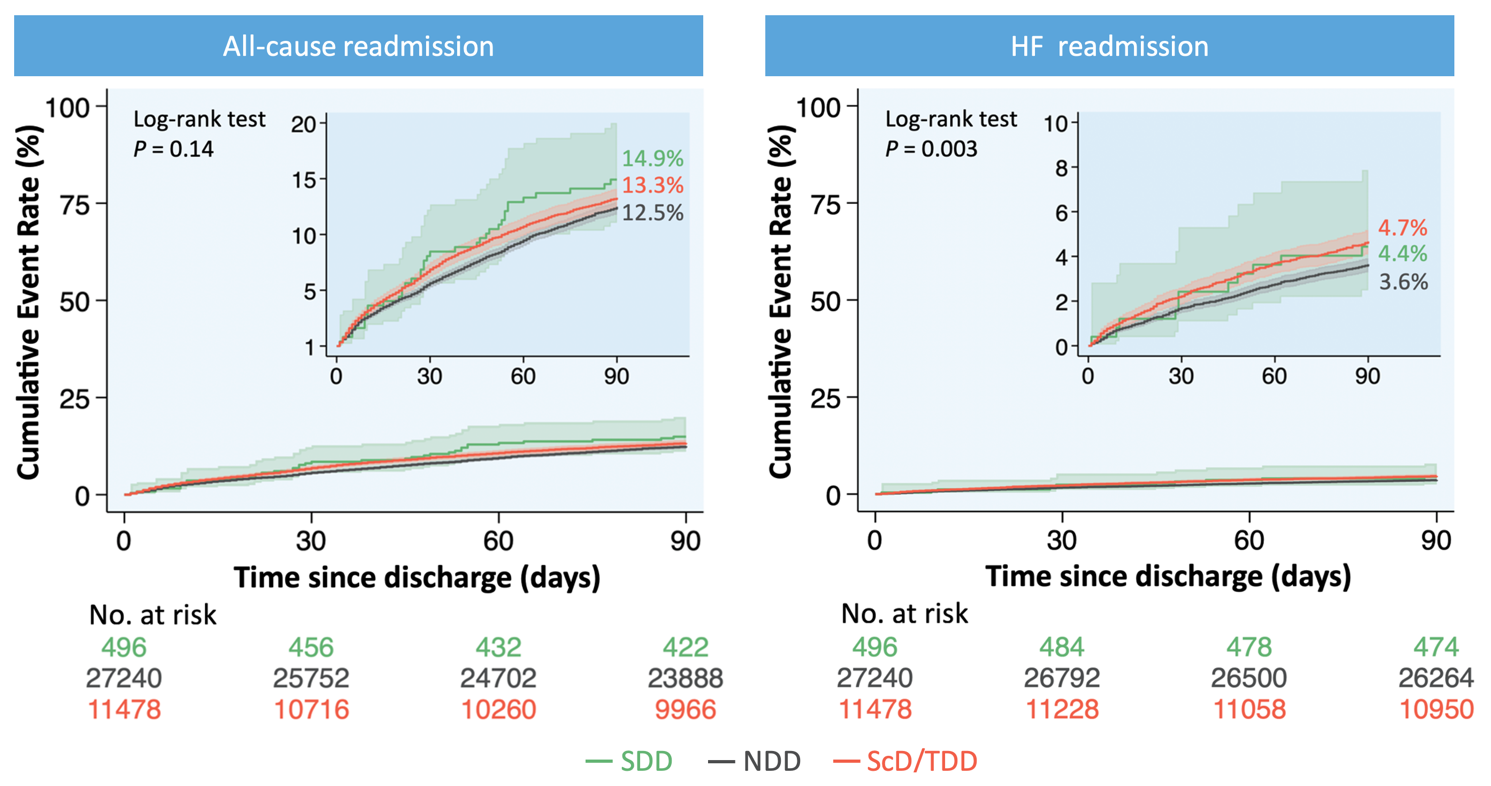


**Supplemental Figure 3.** Love plot showing absolute standardized mean differences (SMDs) for baseline covariates before (blue) and after (red) propensity score matching. The dashed vertical line indicates the prespecified threshold for good balance (SMD = 0.10). CABG=coronary artery bypass grafting; PCI=percutaneous coronary intervention; TIA=transient ischemic attack.


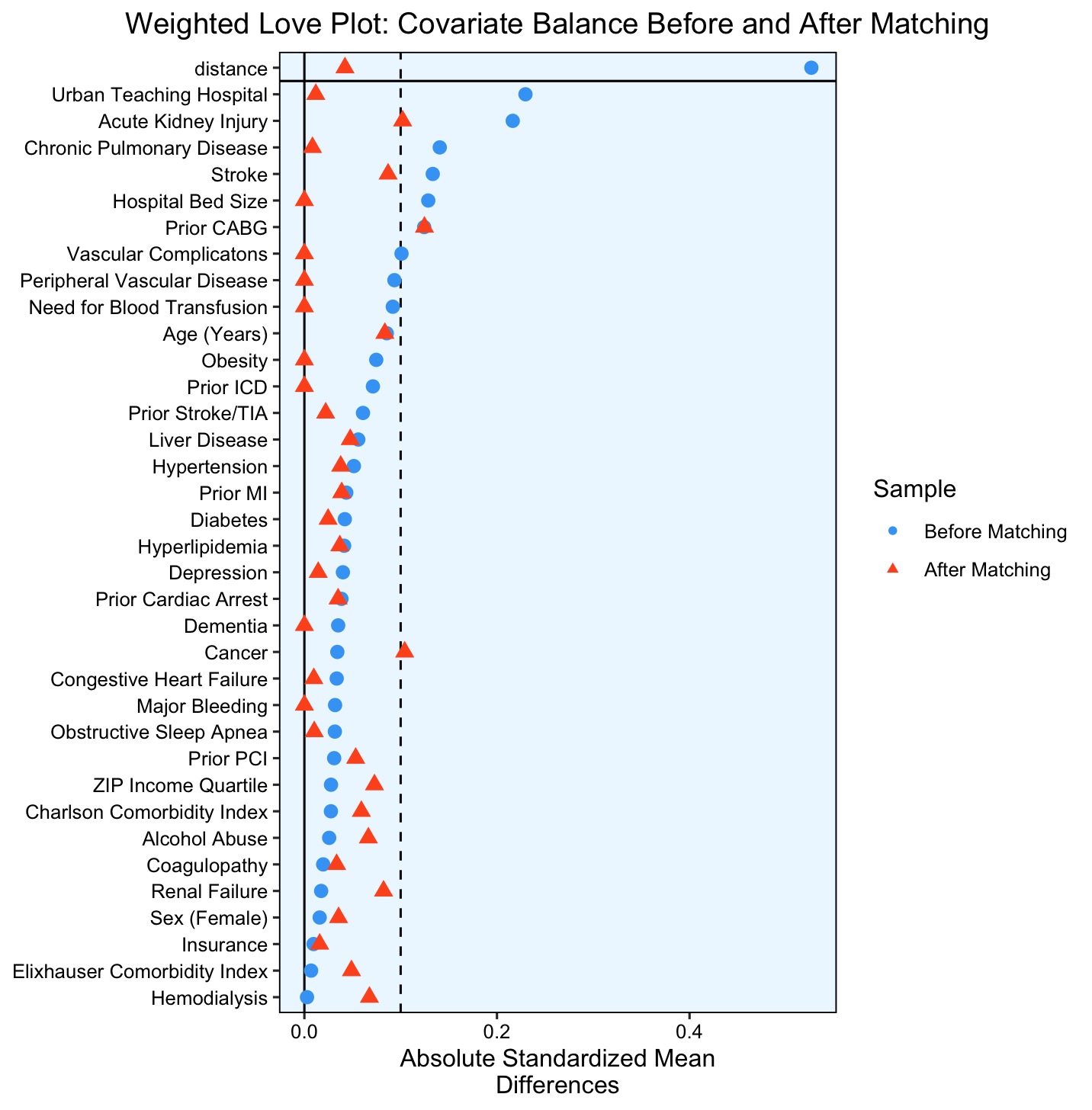


**Supplemental Figure 4.** Kernel density estimates of propensity score distributions for SDD versus NDD before and after propensity score matching. NDD=Next-Day Discharge; SDD=Same-Day Discharge.


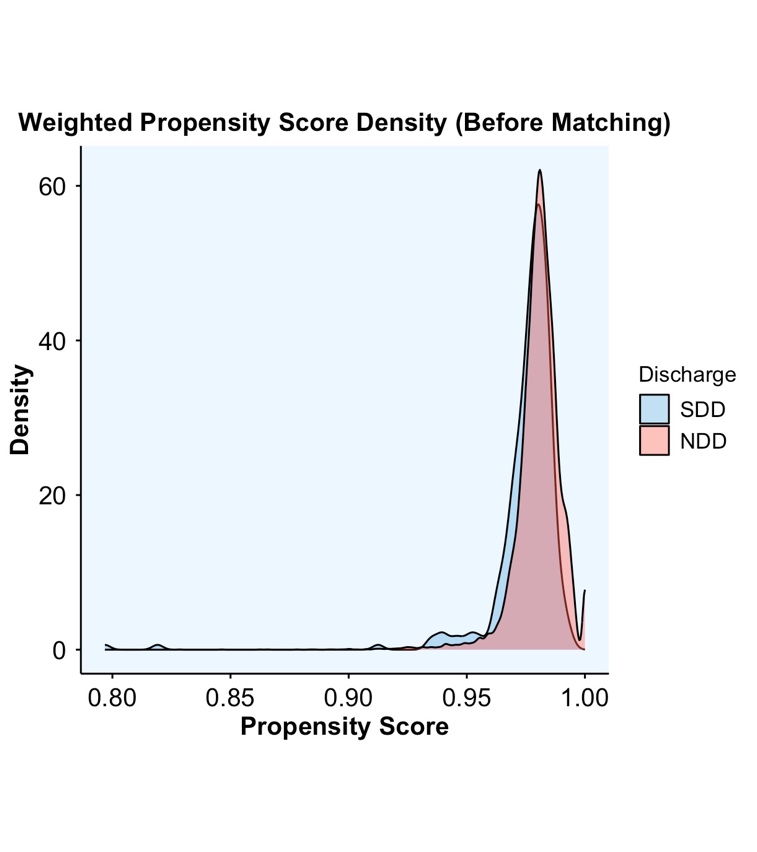

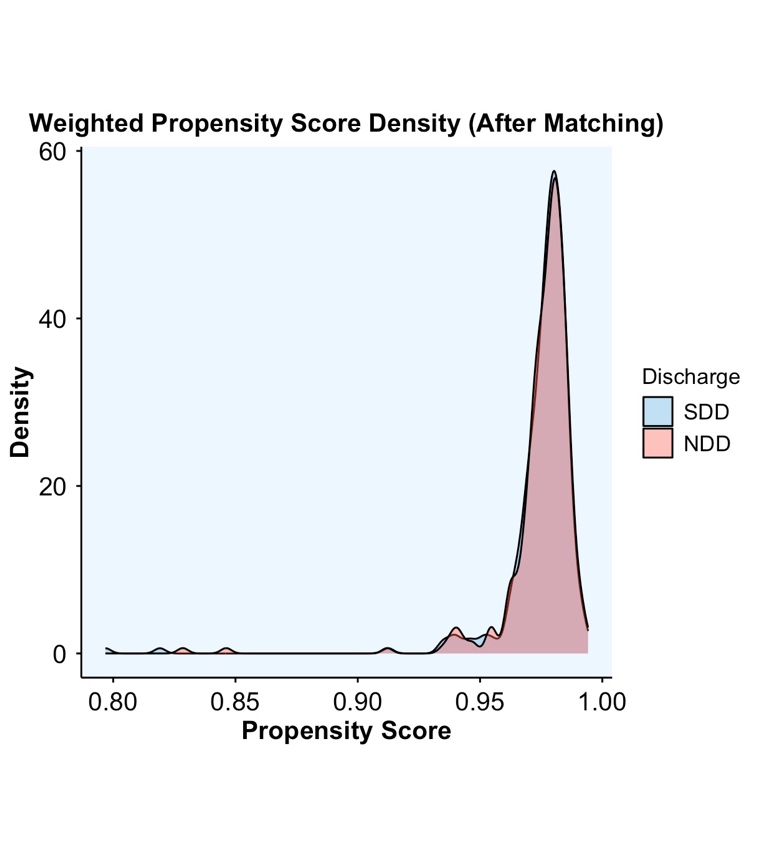

Supplement: Supplemental Tables 1-9 and Supplementary Figures 1-4 [file mmc1.docx]
